# Supplementary material for: Compliance with the WHO-recommended infant feeding practices among HIV-positive mothers at Public Health Facilities of Mekelle, Tigray, northern Ethiopia
Source: PLOS Glob Public Health. 2026 Feb 12;6(2):e0005957. doi: 10.1371/journal.pgph.0005957 (PMC12900308; doi:10.1371/journal.pgph.0005957)
Supplement: Attachment — Submitted filename: Response to reviewers revised version.pdf [file pgph.0005957.s003.pdf]

To Editor-in-Chief

PLOS Global Public Health

Subject: **A rebuttal and point by point response letter that responds to each point raised by the editor and reviewer**

Title: Compliance with the WHO-recommended infant feeding practices among HIV positive mothers at Public Health Facilities of Mekelle, Tigray, norther Ethiopia

Reference Number: PGPH-D-25-01884R1

Round 1: October 31, 2025

Dear Oliver Ian Tolentino

PLOS Global Public Health editor

We are grateful for your observation and constructive comment in protecting participants right that has substantially improved our manuscript.

According to your recommendation we have reviewed the entire study's underlying the data set, (S1 data.sav) looking for any personal identifying data according to Plose guideline. Therefore, here is the step-by-step response letter as follows.

Please let us know if you have any further questions or suggestions.

Sincerely,

Solomon Weldemariam (Corresponding author)

Editor comments:

Comment 1: 1. We note that your Data Availability statement states the following: “We have already provided our data as supporting information file S1 Data.” We also note the following file has been uploaded: “S1 data-second revision.sav”

We noticed that this file contains identifying participant information in COLUMNS K and M (exact dates). Due to the inclusion of this information, we are unable to consider this file for publication and we will remove it from our system.

Response: Dear editor thanks for your concern and recommendation. We have assessed the entire data set for any personal identifying information according to your recommendation. We have also reviewed the journal data policy of your esteemed journal step by step. Therefore, according to your recommendation we have de-identified the specified comments you forwarded including other variables according to the journal guidelines. Kindly see the data set labeled as S1 data. For the improvements we have made.

Comment 2: In light of the possible sharing of personal information, we ask that you please inform your institution’s Institutional Review Board and Data Protection Office/Privacy Office of this incident as soon as possible. Please confirm receipt of this email, and please respond to the following queries by November 5, 2025:

Please confirm that you have reported this incident to your institution’s Institutional Review Board and Privacy Office, and please provide contact details for the official who is responsible for this matter. Please provide a version of the data file containing de-identified information only.

Response: Thank you dear for your concern and recommendation. According to your recommendation we have already tonified the Institutional Review Board of our institution about this incident. We have also attached the de-identified data set in the system.

With regards!

Solomon Weldemariam (corresponding author)
