# Supplementary material for: Compliance with the WHO-recommended infant feeding practices among HIV-positive mothers at Public Health Facilities of Mekelle, Tigray, northern Ethiopia
Source: PLOS Glob Public Health. 2026 Feb 12;6(2):e0005957. doi: 10.1371/journal.pgph.0005957 (PMC12900308; doi:10.1371/journal.pgph.0005957)
Supplement: Attachment — Submitted filename: Response to Reviewers.pdf [file pgph.0005957.s004.pdf]

Response to Reviewers

Journal Name: Plose One Global Public Health

Corresponding author: Solomon Weldemariam

Ref No.: PGPH-D-25-01884R1

Dear Editorial team, Plose One Global Public Health and reviewers of our manuscript, Greetings;

I hope you are doing well.

It has been recalled that we, the authors of this manuscript entitled "Compliance with the WHO-recommended infant feeding practices among HIV-positive mothers at Public Health Facilities of Mekelle, Tigray, northern Ethiopia" (Ref No.: PGPH-D-25-01884R1) submitted for publication in your esteemed journal and received reviewers and editorial comments for the improvement of the manuscript.

In line with this, all authors are very happy with the constructive and invaluable comments given by reviewers as well as the editor. We would also like to thank you for your efforts and time investment in reviewing this manuscript. Accordingly, we have considered all the comments, and we have prepared point-by-point responses and explanations for all the questions raised by the editor and reviewers. Finally, we have submitted all the required documents in their revised form. We hope that we have addressed all the questions raised by the reviewers and editor. However, if you have any points which need further clarity and explanation, let us know and we are ready to address any concerns.

Note for reviewers and editors: The given page and line numbers below in this point-by-point response to reviewers corresponds with the clean manuscript.

Thank you again for your precious time and energy!

Editor concern:

Recognition and concern: Thank you for submitting your manuscript to PLOS Global Public Health. After careful consideration, we feel that it has merit but does not fully meet PLOS Global Public Health's publication criteria as it currently stands. Therefore, we invite you to submit a revised version of the manuscript that addresses the points raised during the review process.

Response: Thank you, dear, for your recognition that our manuscript is worth publishing in your esteemed journal given that we are able to improve the manuscript in accordance with the journal requirements. Therefore, as much as possible, we have tried to address all the concerns and comments of reviewers' as well as the editor.

Comment: Please respond to both reviewers and if you feel like there is overlap in their comments, feel free to reference your response across reviewer comments.

Response: Thank you, editor, we have responded to reviewers' comments as recommended

Recommendation: Please include the following items when submitting your revised manuscript:

A letter that responds to each point raised by the editor and reviewer(s). You should upload this letter as a separate file labeled 'Response to Reviewers'.

A marked-up copy of your manuscript that highlights changes made to the original version. You should upload this as a separate file labeled 'Revised Manuscript with Track Changes'.

An unmarked version of your revised paper without tracked changes. You should upload this as a separate file labeled 'Manuscript'.

Response: Thank you, for your recommendation, and we have already labeled the submitted files accordingly.

Recommendation: Guidelines for resubmitting your figure files are available below the reviewer comments at the end of this letter.

Response: Thank you, editor, and we have prepared our figure file based on your recommendation using NAAS.

Journal Requirements:

If the reviewer comments include a recommendation to cite specific previously published works, please review and evaluate these publications to determine whether they are relevant and should be cited. There is no requirement to cite these works unless the editor has indicated otherwise.

Response: Thank you dear for your constructive comment. However, the is no such comment from the reviewers and all our references are available on line.

Please review your reference list to ensure that it is complete and correct. If you have cited papers that have been retracted, please include the rationale for doing so in the manuscript text, or remove these references and replace them with relevant current references. Any changes to the reference list should be mentioned in the rebuttal letter that accompanies your revised manuscript. If you need to cite a retracted article, indicate the article's retracted status in the References list and also include a citation and full reference for the retraction notice.

Response: We appreciate your valid concern, and we have checked this issue, but all our references are complete and correct. Moreover, all the references we used are available online there is not retracted reference. NB. We have made changes only on reference number 6.

Reviewers' comments:

Reviewer's Responses to Questions

Comments to the Author

1. If the authors have adequately addressed your comments raised in a previous round of review and you feel that this manuscript is now acceptable for publication, you may indicate that here to bypass the "Comments to the Author" section, enter your conflict-of-interest statement in the "Confidential to Editor" section, and submit your "Accept" recommendation.

Reviewer #1: (No Response)

Reviewer #2: (No Response)

2. Does this manuscript meet PLOS Global Public Health's publication criteria? Is the manuscript technically sound, and do the data support the conclusions? The manuscript must describe methodologically and ethically rigorous research with conclusions that are appropriately drawn based on the data presented.

Reviewer #1: Yes

Reviewer #2: Yes

3. Has the statistical analysis been performed appropriately and rigorously?

Reviewer #1: Yes

Reviewer #2: Yes

4. Have the authors made all data underlying the findings in their manuscript fully available (please refer to the Data Availability Statement at the start of the manuscript PDF file)?

The PLOS Data policy requires authors to make all data underlying the findings described in their manuscript fully available without restriction, with rare exception. The data should be provided as part of the manuscript or its supporting information, or deposited to a public repository. For example, in addition to summary statistics, the data points behind means, medians and variance measures should be available. If there are restrictions on publicly sharing data—e.g. participant privacy or use of data from a third party—those must be specified.

Reviewer #1: Yes

Reviewer #2: Yes

Response: We would like to thank the reviewers, for your recognition and acknowledging our work and efforts.

5. Is the manuscript presented in an intelligible fashion and written in standard English?

PLOS Global Public Health does not copyedit accepted manuscripts, so the language in submitted articles must be clear, correct, and unambiguous. Any typographical or grammatical errors should be corrected at revision, so please note any specific errors here.

Reviewer #1: No

Thank you!

Reviewer #2: Yes

Response: Dear reviewer #2, thank you for your meticulous observation of sentences and paragraphs that need a typographical and Grammar edition. We have addressed these issues accordingly, and we have indicated the improvements made to page and line numbers as described under Reviewer #2 comments and the step-by-step response:

6. Review Comments to the Author

Please use the space provided to explain your answers to the questions above. You may also include additional comments for the author, including concerns about dual publication, research ethics, or publication ethics. (Please upload your review as an attachment if it exceeds 20,000 characters)

Recognition from: Reviewer #1: Thank you very much for giving me the opportunity to review this paper. Despite all the strides made in the control of HIV, it continues to be a significant burden on healthcare systems, communities and individuals. I found this paper to be an important contribution to the existing body of work on PMTCT. Please note that my comments are based on the revised version of the paper that is in tracked changes.

Response: Dear Reviewer #1: Thank you for recognizing and approving being publishing this manuscript in this journal can add updated information to the scientific community which leads to improvement of PMTCT and feeding option services.

General

Concern/comment: I found the paper to be well structured. However, I believe that the quality and readability of the paper will be greatly strengthened by having a thorough review of the language. For example, the lines 102-104 may read better if presented as "Hence, to create a HIV-free generation, countries need updated and consistent information as well as updated strategies on infant feeding practices among HIV- exposed infants." Throughout the paper, there are many grammatical errors and issues with syntax.

Response: Dear Reviewer #1: Thank you for recognizing about the structure of our manuscript and constructive comments. Your comment is valid, and we have addressed it accordingly. Kindly see the improvements made on #page 5 #line number 108-110.

Background

Comment: Line 88-91. This sentence seems to be incomplete. The factors have been enumerated but what are these factors associated with precisely?

Response: Really, we thank you for your constructive comments and we have considered your comments. Kindly see the revision made on #page 4-5 #line numbers 91-97.

Comment: Sample size determination and sampling process

Comment: - Line 137-138: There is no need to put “41.3% vs 21.5%, respectively” in brackets.

Response: Thank you dear for your meticulous observation, and we have addressed your comment accordingly. Kindly see the amendment made under Sample size determination sub heading, on #page 7 #line number 144

Comment: - Line 138-142: Here, you mention seven factors associated with infant feeding practice. It will be helpful if these seven factors are listed because prior to this point in the paper, you did not specifically list these seven factors that were of particular interest to you.

Response: Thank you dear for your valid comment, and we have listed the variables accordingly. Kindly see the amendment made on #page 7 #line number 145-148

Results

Concerns and comments: - Line 288: Please consider adding the unit to the mean infant age. Should this be 9 months?

Response: Thank you dear! You are right. The unit of measurement is months. So, we have revised accordingly and kindly see the revision made on #page 13 #line number **276**

- Table 4: Episiotomy is not a form of delivery. Seeing that this was the only place it was mentioned in the paper, the authors should consider removing it from the table.

Response: Thank you dear! your comment is valid and accepted. Kindly see the revision made on table 4 found in #page 20

Discussion

Comment/concern: - The use of the word “discrepancy” when comparing the results of this study with other studies (e.g. lines 435 and 449) may not necessarily be correct. I believe that the results of your study may be different from other studies but shouldn’t be taken as a discrepancy. The authors may wish to consider changing the use of the term discrepancy to perhaps difference.

Response: Thank you dear, we have changed the word “discripance” into difference according to your recommendation including in other paragraphs. Would you kindly check the revision made on #page 21,22 #line numbers 384 and 385, #page 22, line numbers, 389, 391, 397, #page 24, line number 439.

Response to reviewer#2

Reviewer #2: The revised version of the manuscript is nearly perfect. There are few areas which I would like to request the authors to work on as follows:

ABSTRACT

Comment: Line 28 - I suggest to do the following:

o Edit “Compliance” to start with a small “c”; and

o Write WHO in its long form and the abbreviation in brackets as follows; “World Health Organization (WHO)”.

Response: Thank you dear for your interesting comments, and we have corrected them as per your recommendation. Kindly check the correction on #page 2 #line number 29

Comment: Line 36 – I suggest to replace “world health organization” with its abbreviation “WHO”.

Response: Thank you dear for your interesting comment, and we have corrected it as per your recommendation. Kindly check the correction on #page 2 #line number 37

BACKGROUND

Comment/recommendation: Line 49 – I suggest to write HIV in its long form and the abbreviation in brackets as follows; “Human Immunodeficiency Virus (HIV)”.

Response: Thank you dear for your interesting comment, and we have corrected it as per your recommendation, but this is done in the abstract section first line. Kindly check the correction on first line of the abstract #page2 #line number 25

Comment: Line 51 – I suggest to replace "“Human Immunodeficiency Virus (HIV)” with its abbreviation “HIV”.

Response: Thank you for your comment, and we have corrected it as per your recommendation. Kindly check the correction on #page 3 #line number 53

Comment: Line 62 – I suggest to edit “UNAID” to be “UNAIDS”.

Response: Thank you dear for your comment, and we have corrected it as per your recommendation. Kindly check the correction on #page 3 #line number 64

Comment: Line 75 – I suggest to write ART in its long form and the abbreviation in brackets as follows; “antiretrovirals therapy (ART)”.

Response: Thank you dear for your interesting comment, and we have corrected it as per your recommendation. Kindly check the correction on #page 4 #line number 77

Comment: Line 80– I suggest to edit “also associates” to be “is also associated”.

Response: Thank you dear for your valid comment, and we have addressed this issue as per your recommendation. Kindly check the correction on #page 4 #line number 81-82

Comment: Lines 84-85– I suggest to insert “other” between “from & regions”.

Response: Thank you for your constructive comment, and we have corrected it as per your recommendation. Kindly check the correction on #page 4 #line number 88

Comment: Line 89 – I suggest to write ANC in its long form and the abbreviation in brackets as follows; “antenatal care (ANC)”.

Response: Thank you dear for your interesting comment, and we have corrected it as per your recommendation in the abstract section. Kindly check the correction on #page 2 #line number 38

Comment: Line 102 – I suggest to edit “update” to be “updated”.

Response: Thank you dear for your interesting comment, and this was also the suggested comment from reviewer #1. So, we have corrected it as per your recommendation. Kindly check the correction on #page 5 #line number 109

Comment: Line 105 – I suggest to: (a) edit “update” (between Moreover & information) to be “updated; and (b) insert “is” between “Tigray & also”.

Response: Thank you dear for your interesting comment, and this was also suggested by reviewer #1. So, we have corrected it as per your recommendation. Kindly check the correction on #page 5 #line number 111

METHODS

Study setting, design and period

Comment: Line 118 – I suggest to insert "/" between HIV and AIDS.

Response: Thank you for your comment, and we have corrected this issue. Kindly check the correction on #page 6 #line number 125

DISCUSSION

Comment: Line 381 – I suggest to edit "vary" (between "be & from") to be varying".

Response: Thank you dear your comment is acceptable, and we have corrected it accordingly. Kindly check the correction on #page 22 #line number 390

CONCLUSION AND RECOMMENDATION

Comment: Line 507 – I suggest to insert "are" between HIV & still.

Response: Thank you for your meticulous observation, and we have corrected it accordingly. Kindly check the correction on #page 27 #line number 519

Comment: Line 514 – I suggest to replace "caesarean section" with its abbreviation "CS".

Response: We appreciate your interesting comment, and we have addressed it accordingly. Kindly check the correction on #page 28 #line number 526

Figure Resubmissions:

Recommendation: While revising your submission, we strongly recommend that you use PLOS's NAAS tool (https://ngplosjournals.pagemajik.ai/artanalysis) to test your figure files. NAAS can convert your figure files to the TIFF file type and meet basic requirements (such as print size, resolution), or provide you with a report on issues that do not meet our requirements and that NAAS cannot fix.

Response: Thank you for your recommendation, and we have applied NAAS tool accordingly. Please kindly check Figure 1.

NB. Dear reviewers and editor, this is to inform you that, in addition to the improvements made indicated with page number and lines number, we have also made a lot of language editions throughout the manuscript. So, kindly please check the manuscript, particularly the revised manuscript with track changes.

Thank you again!!
